# Effects of Corrective Exercises on Lumbar Lordotic Angle Correction: A Systematic Review and Meta-Analysis

**DOI:** 10.3390/ijerph19084906

**Published:** 2022-04-18

**Authors:** Vanja Dimitrijević, Tijana Šćepanović, Vukadin Milankov, Miroslav Milankov, Patrik Drid

**Affiliations:** 1 Faculty of Sport and Physical Education, University of Novi Sad, 21000 Novi Sad, Serbia; dimitrijevicvanja@gmail.com (V.D.); tijanascepanovic021@gmail.com (T.Š.); 2 Faculty of Medicine, University of Novi Sad, 21000 Novi Sad, Serbia; vukadin.milankov@mf.uns.ac.rs (V.M.); miroslav.milankov@mf.uns.ac.rs (M.M.); 3 Institute for Children and Youth Health Care of Vojvodina, 21000 Novi Sad, Serbia; 4 Department of Orthopedic Surgery and Traumatology, Clinical Center of Vojvodina, 21000 Novi Sad, Serbia

**Keywords:** lordosis, hyperlordosis, low back pain

## Abstract

Lumbar lordosis is one of the most important parts of the spine, which is of special importance due to its unique position and direct contact with the pelvis. The aim of this study was to combine the results of several studies and to evaluate the magnitude of the effect of different Lumbar lordotic angle correction programs through meta-analysis. This study has been developed in accordance with the Preferred Reporting Items for Systematic Reviews and Meta-Analyses (PRISMA) Statement. Four databases were searched for articles collection: PubMed, Cochrane Library, Web of Science, and Google Scholar. The key search terms were: “Lumbar Lordotic angle”, “Lordosis”, “Hyperlordosis”, “Corrective exercise”, and “Low back pain. “The articles included in our study were limited to original articles written only in English that met the following inclusion criteria: (1) participants with lumbar lordosis or hyperlordosis or low back pain; (2) different programs of corrective exercises were applied; (3) Lumbar lordotic angle used as outcome measures. Ten studies are included in our systematic review and meta-analysis. The effect size for the Lumbar lordotic angle outcome was (SMD = 0.550, *p ˂* 0.001, moderate effect size). Subgroup analysis for Lumbar lordotic angle: Subgroup Younger group (SMD = 0.640, *p ˂* 0.001), Subgroup Older group, (SMD = 0.520, *p ˂* 0.001). Subgroup Treatment (SMD = 0.527, *p ˂* 0.001), Subgroup No treatment (SMD = 0.577, *p* = 0.002). This was the only outcome assessed in our analysis. The current meta-analysis indicates that different correction methods have a positive effect on subjects with lumbar lordosis or hyperlordosis. In the following research, we should try to determine which corrective methods have the best effects.

## 1. Introduction

Back pain is a common condition that restricts movement and activities of daily living [1]. In addition, reduced physical activity due to industrialization has consequently increased lower back pain [2]. With prolonged back pain, chronic lower back pain leads to pain, hypoesthesia, and decreased strength and endurance of deep spinal muscles under the muscular system and sensory-motor control, affecting the daily lives and social activities of individuals [3,4]. Lumbar lordosis is one of the most important parts of the spine, which is of special importance due to its unique position and direct contact with the pelvis. Lumbar lordosis serves to provide strength against the compression forces of gravity [5,6]. Normal lumbar lordosis protects the posterior spinal ligament system from excessive stress [7] and acts as a shock absorber during sudden applied vertical forces [8]. Increased lordosis is advocated as a major cause of postural pain, radiculopathy, and facet pain [9,10]. Excessive lordosis leads to increased compression of the apophyseal joint and increased anterior shear force at the lumbosacral joint [11,12].

Special attention should be paid to the spine in order to have a good physical condition [13]. In addition to bones, ligaments, muscles, and vertebral discs play a key role in the formation of lordosis. Without muscle action, pelvic girdle performance does not have sufficient stability [14]. Central stabilization of the spine is supported by special muscles such as multifidus, transversus abdominis, and internal muscles in the trunk. These muscles act late in patients with hyperlordosis [15]. Muscles provide vertebral stability in focal shape [14]. Weakness in any of the muscles of the lumbar-pelvic girdle can accompany pelvic rotations and deflections of the posterior arch, disturbing muscle balance in this area [16], and thus a person may be prone to musculoskeletal disorders [17]. Pelvic muscle balance is one of the factors influencing lordosis [18]. For the functional position of the central part of the body, the mobility of the hip joint on the one hand and the stability of the trunk, on the other hand, are important. Coordination between all torso and thigh muscles is crucial to control and maintain a normal spine position where no specific muscle is involved in increasing core stability. The balance between the muscles on the four sides of the spine determines the stability of the spine [19]. There are various factors that affect lumbar lordosis.

Some studies have shown that the extent of lumbar lordosis is affected by age and gender, pregnancy, or obesity [20,21]. To improve spinal segment instability, lumbar stabilization exercises that strengthen the local muscle group located deep in the trunk around the lumbar vertebrae, which play an important role in ensuring dynamic stability of the spinal segments, are useful for alleviating functional spinal disability [22,23]. Lumbar stabilization is important for maintaining the spine and performing limb movements [24] and is used to adjust the imbalance between the abdominal and trunk extensor muscles. Such an imbalance causes diseases in the lumbar vertebrae of the musculoskeletal system, which must be treated to prevent lower back pain [25]. Numerous treatments such as rest, exercise therapy, traction, osteopathic therapy, manipulative therapy, massage, mobilization, and electrotherapy have been reported to treat lower back pain. However, there is still no general consensus on which method is more effective in treating lower back pain [26]. On the one hand, researchers have shown that more than 90% of patients improve after six months of participation in a hydrotherapy program, and according to this report, hydrotherapy is an effective treatment for patients with lower back pain [27,28]. Exercises are recommended as good for lumbar lordosis Williams and McKenzie [26]. The sling exercise, which is based on the concept of lumbar stabilization, is also used to treat back pain. The sling exercise is a closed-chain exercise with a load, using a suspension that is said to improve lumbar curvature and muscle imbalance [29,30,31].

Lumbar lordotic angle (LLA) is measured by simple X-rays of the lateral view of the lumbar region, at the intersection between the line extending from the upper plate L1 and the other extending from the lower plate L5 [32]. Skaf et al. [33] report the determination of Lordotic angle using the Cobb method using Magnetic Resonance Imaging (MRI). The images are read using as reference L1 and S1 vertebrae of the body, where lines are drawn along the upper end of the plate to extend past the vertebral body. Perpendiculars are then added on the convergence side of the two lines, and the angle of intersection of these two lines is measured, forming a Cobb angle, thus giving a global estimate of lumbar lordosis. LLA can also be measured using a Flexi curve ruler. Participants stand barefoot on the cardboard where the position of the feet is marked. The ruler is placed between the twelfth thoracic vertebra (T12) and the sacrum (S1). The curvature of the ruler is then drawn on paper. The vertical line is then drawn from T12 to S1 to measure the maximum length (L) and depth (H) of the lumbar region in centimeters to determine the LLA using the Spinal Mouse [34,35]. Spinal Mouse is a device that, in combination with a computer program, estimates the curves of the spine without the use of harmful radiation. The normal angle of lordosis is 30°, and angles > 40° indicate hyperlordosis [36].

The aim of this study was to combine the results of several studies and to evaluate the magnitude of the effect of different LLA correction programs through meta-analysis. Combining the results of two or more studies is a great advantage when concluding on the application of certain treatments, more so than when we draw conclusions based on only one study.

## 2. Materials and Methods

### 2.1. Study Design

This paper has been developed and reported in accordance with the Preferred Reporting Items for Systematic Reviews and Meta-Analyses (PRISMA) guidelines [37].

### 2.2. Data Sources and Search Strategy

**Figure 1:**
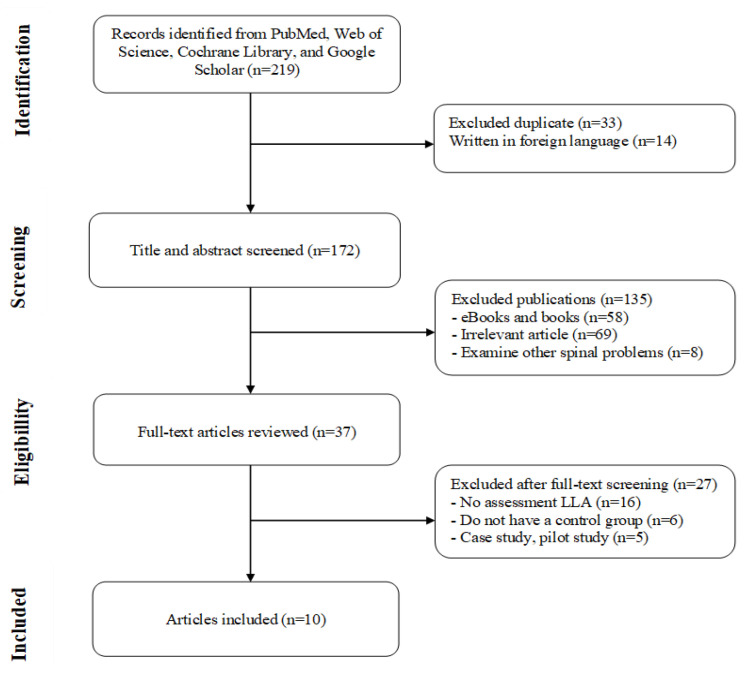
Flow diagram of the process of study selection for the meta-analysis.

A search strategy was developed to identify all relevant studies that evaluate the effect of different corrective exercises in the treatment of lordosis and hyperlordosis. Our systematic search included PubMed, Web of Science, Cochrane Library, and Google Scholar databases. We used combinations of the subject titles “Lumbar Lordotic angle”, “Lordosis”, “Hyperlordosis”, “Corrective exercise”, and “Low back pain.” The search strategy is shown in Figure 1. We also manually searched for reference citations of identified critiques and selected original research articles to download the full text.

### 2.3. Study Selection

PICOS (Population, Interventions, Comparators, Outcomes, Study Designs) eligibility criteria described in PRISMA were adopted for inclusion/exclusion of the studies [37]. (1) P (population) individuals diagnosed with lordosis or hyperlordosis or low back pain, (2) I (intervention) utilized some form of the corrective exercises, (3) C (comparison) control group defined as no treatment, standard care, or other corrective programs, (4) O (outcome) Lumbar lordotic angle expressed in degrees was the only outcome at which the magnitude of the effect was measured, (5) S (study design) prospective studies with controls that were published in or after 2010 were included. The inclusion of studies in our analysis was limited to English only. Studies excluded were systematic reviews, meta-analyses, study protocols, books, eBooks, book reviews, case studies, pilot studies, and conference publications. A systematic search of four electronic databases (PubMed, Web of Science, Cochrane Library, and Google Scholar) was conducted in August 2021. The inclusion/exclusion of studies was done by two researchers by consultation and consensus.

### 2.4. Data Extraction and Quality Assessment

After selecting studies based on inclusion and exclusion criteria, the two investigators independently conducted data extraction. The following variables are abstracted into a pre-formatted table: authors, year of publication, characteristics of study participants (number of participants, age), program type, outcomes, lordotic angle size, sessions per week, duration, and instrument to measure an angle. Nine of the included studies had available data, while the study by Okhli et al. [34] used two experimental groups and one control group for which no data were available. Two researchers independently assessed the quality of the studies involved. The risk of bias was assessed for each study using the Cochrane Risk Bias Tool [38], which assesses seven sources of bias, including randomization, allocation concealment, blinding of participants and personnel, blinding of outcome assessment, completeness of outcome data, selective outcome reporting, and other potential bias. Each study based on the above seven aspects was examined and subsequently assessed as low risk, high risk, or unclear risk.

### 2.5. Data Synthesis and Analysis

Meta-analysis and statistical analysis were performed using Meta-Analyst software (Brown University) [39]. The magnitude of the effect was estimated for the outcome of the Lumbar lordotic angle. For each study, standardized mean difference (SMD) and 95% confidence intervals (CI) were calculated for continuous outcomes, a random model. For the Lumbar lordotic angle outcome, the effect size was measured post minus before intervention in the experimental and control groups. 

According to Cohen’s guide, values of ≥0.2, ≥0.5, and ≥0.8 show small, medium, and large effect sizes, respectively [40]. After that, the analysis of the subgroup according to the factor for years and the analysis of the subgroup according to the factor of the control group (treatment or no treatment) were performed in order to separately assess the magnitude of the effect and solve this problem in case of increased heterogeneity. *p* < 0.05 was considered statistically significant. Heterogeneity between studies was assessed using the Higgins I² test and *p* values. The guide for interpreting heterogeneity in the meta-analysis of randomized trials is as follows: 0% to 40%: might not be important; 30% to 60%: may represent moderate heterogeneity; 50% to 90%: may represent substantial heterogeneity; 75% to 100%: considerable heterogeneity.

Based on the search strategy, a total of 219 studies were selected from the initial database search. Of that number, 33 studies were excluded because of duplication, and also excluded were 14 studies that were not written in English; therefore, 172 studies were selected for further analysis. After the abstract and title were displayed, 135 studies were excluded because they did not meet the inclusion criteria. The remaining 37 studies were reviewed in full. When the full-text articles were reviewed, 27 studies were excluded. The remaining 10 studies are included in this review article and meta-analysis. The flow diagram of the study selection process is shown in Figure 1.

Table 1 shows the main characteristics of the included studies. A total of 482 respondents participated in the ten included studies, and the sample size of the included studies ranged from 29 to 154. The age of the respondents ranged between nine and 60 years old. The total length of treatment ranged from two weeks to 12 weeks. Studies by Yakhdani et al. [41] and Ko et al. [32] used two experimental groups and one control group. In our meta-analysis, we used the results of both experimental groups in the study of Ko et al. [32] and one in the study of Yakhdani et al. [41]. The careful review found that studies by Fatemi et al. [42] and Javid et al. [43] used the same cohort and that the outcome results were the same. For this reason, a Leave-one-out meta-analysis was performed to decide whether to exclude one of these two studies from the meta-analysis.

### 2.6. Medicine Risk of Bias

**Figure 2:**
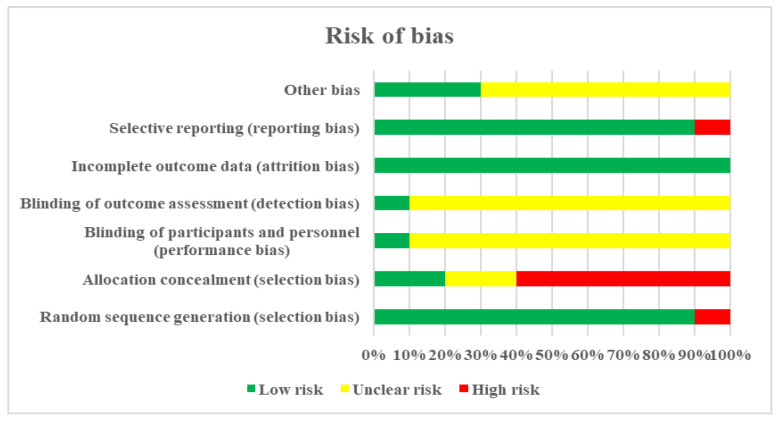
Risk of bias graph: review authors’ judgments about each risk of bias item.

**Figure 3:**
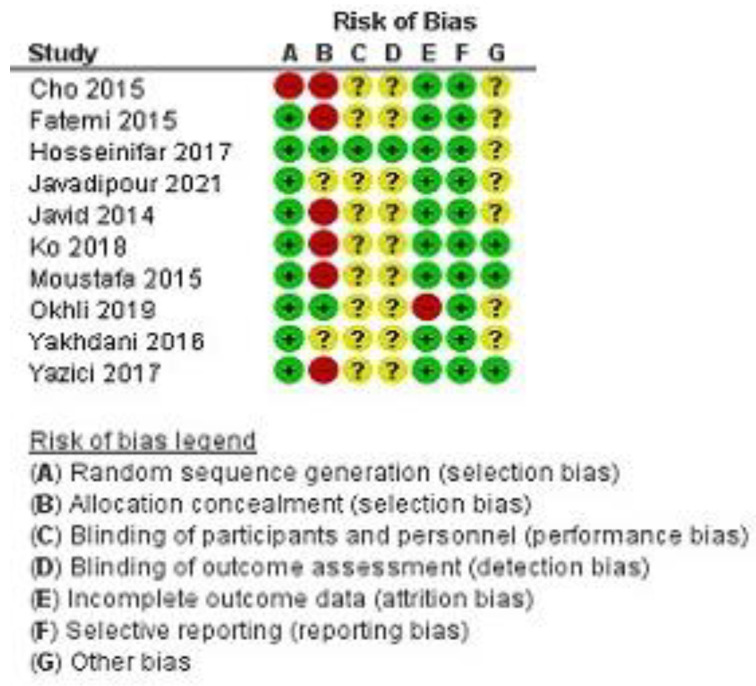
Risk of bias for each study: green—low risk, yellow—unclear risk, red—high risk.

Figure 2 and Figure 3 present the summary of the bias risk for each study included. For the item of “Random sequence generation”, nine studies used randomization, while the study of Cho et al. [44] does not provide information on the process of selecting respondents. Allocation concealment was low risk in two studies, unclear risk in two studies, and high risk in six studies. Due to the nature of the intervention, participants and experimenters could not be blinded to the treatment, but one study reports that it used blinding. For item “Blinding of outcome assessment”, one study adopted a single-blind method to evaluate the intervention measures. For item “Selective reporting” study, Okhli et al. [34] have available data for two experimental groups and unavailable data for the control group. Due to the objective outcome measures, outcome data were considered low risk in 10 studies.

## 3. Results

### 3.1. Meta-Analysis

#### 3.1.1. Lumbar Lordotic Angle

**Figure 4:**
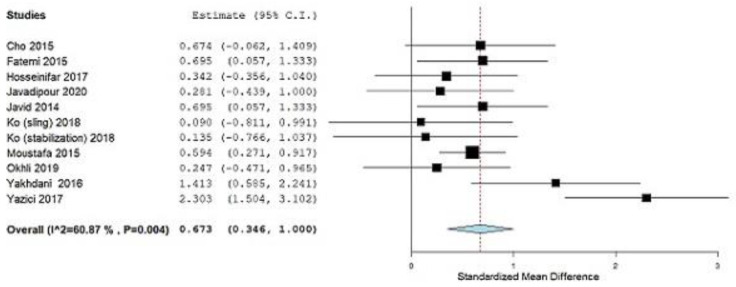
Standardized mean difference (SMD), outcome: Lumbar lordotic angle. Squares represent the SMD for each trial. Diamonds represent the pooled SMD across trials.

The only outcome assessed in our study was LLA. LLA is the most commonly used value for quantifying lordotic spinal deformity [42,44]. All ten studies included in our meta-analysis used the LLA as the outcome of their measurement. After data pooling, statistical significance was achieved (SMD = 0.673; 95% CI = 0.346, 1.000, *p ˂* 0.001), heterogeneity (I^2^ = 60.87%, *p* = 0.004) (Figure 4). Due to substantial heterogeneity (I^2^ = 60.87%), a decision was made by the consensus of two researchers to exclude the study by Yazici et al. [35] from further analysis, assuming that it causes substantial heterogeneity which according to the analysis can be seen in Figure 4.

**Figure 5:**
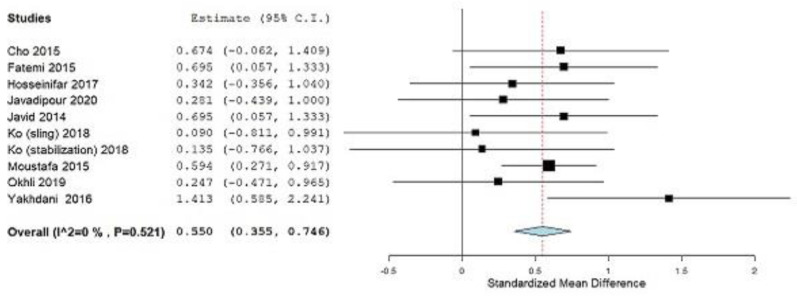
Standardized mean difference (SMD), without study Yazici et al. [35] Outcome: Lumbar lordotic angle. Squares represent the SMD for each trial. Diamonds represent the pooled SMD across trials.

After this decision, the following results were obtained: (SMD = 0.550; 95% CI = 0.355, 0.746, *p ˂* 0.001), heterogeneity (I^2^ = 0%, *p* = 0.521) (Figure 5). 

**Figure 6:**
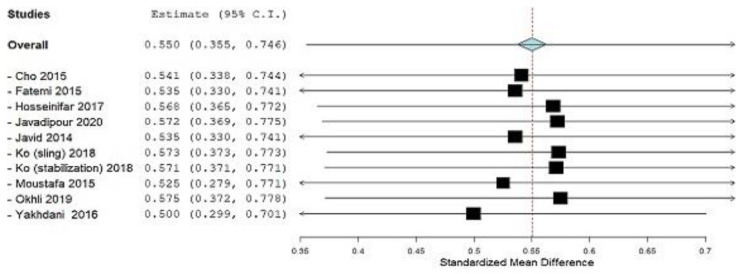
Leave-one-out meta-analysis, outcome: Lumbar lordotic angle.

Figure 6 shows the Leave-one-out meta-analysis for the outcome of the Lordotic angle that was done to decide whether to exclude one of the studies Fatemi et al. [42] and Javid et al. [43] from further analysis. The results show that nothing significant would change (SMD = 0.550 vs. SMD = 0.535). For the study by Okhli et al. [34], one of the experimental groups was taken as a control group in the subgroup “Treatment” because the data of the control group were not available.

#### 3.1.2. Subgroup Analysis

**Figure 7:**
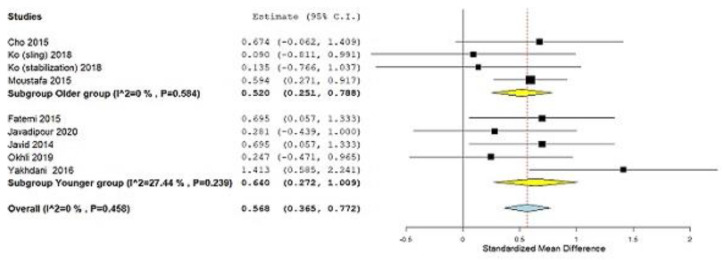
Standardized mean difference (SMD), outcome: Lumbar lordotic angle, Subgroup analysis, Older and Younger group. Squares represent the SMD for each trial. Diamonds represent the pooled SMD across trials.

**Figure 8:**
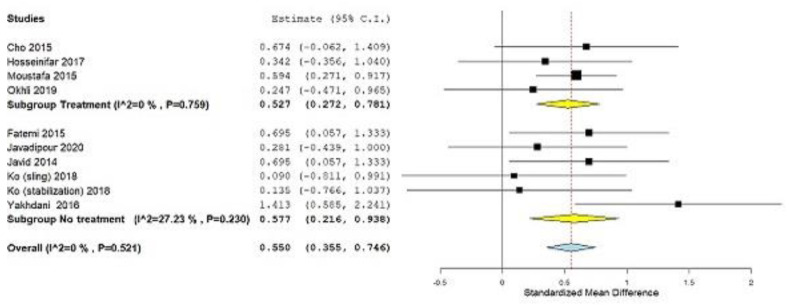
Standardized mean difference (SMD), outcome: Lumbar lordotic angle, Subgroup analysis, Control group Treatment or No treatment. Squares represent the SMD for each trial. Diamonds represent the pooled SMD across trials.

After a detailed analysis, the analysis of the subgroup for the factor of the year was performed and the following results were obtained: (Subgroup Younger group, SMD = 0.640; 95% CI = 0.272, 1.009, *p ˂* 0.001, heterogeneity I^2^ = 27.44%, *p* = 0.239; Subgroup Older group, SMD = 0.520; 95% CI = 0.251, 0.788, *p ˂* 0.001, heterogeneity I^2^ = 0%, *p* = 0.584) (Figure 7). Subgroup analysis was also performed for the control group factor (treatment or no treatment) and the following results were obtained: (Subgroup Treatment, SMD = 0.527; 95% CI = 0.272, 0.781, *p ˂* 0.001, heterogeneity I^2^ = 0%, *p* = 0.759; Subgroup No treatment, SMD = 0.577; 95% CI = 0.216, 0.938, *p* = 0.002, heterogeneity I^2^ = 27.23%, *p* = 0.230) (Figure 8).

## 4. Discussion

### 4.1. Summary of Main Results

In this systematic review, we combined the results of 10 studies to calculate the magnitude of the effect of corrective exercise on subjects with lumbar lordosis and hyperlordosis, of which there were 482, which was the aim of this study. Correction and prevention of lumbar lordotic deformities is the main goal of therapists who work with subjects who have lumbar lordosis and hyperlordosis. The only outcome for which we determined the magnitude of the effect of corrective exercises was LLA. For this outcome, subgroup analysis for the year factor and for the control group factor (treatment or no treatment) was performed. In subjects with lumbar lordosis or hyperlordosis, LLA is used as a guideline for physiotherapists to set goals and plans for further interventions [41,43]. By applying the different corrective exercise approaches shown in Table 1, the LLA was statistically significantly reduced. Statistical analysis of LLA outcomes for the total effect size of corrective exercises achieved statistical significance (SMD = 0.550, *p ˂* 0.001, according to the Cohen guide, is a moderate effect size) (Figure 5). Previously, the study by Yazici et al. [35] was excluded from the analysis because it contributed to substantial heterogeneity (I^2^ = 60.87%, *p* = 0.004), as seen in (Figure 4). After that, there was no more heterogeneity (I^2^ = 0%, *p* = 0.521) (Figure 5). Leave-one-out meta-analysis shows how each individual study affects the overall assessment of other studies (Figure 6).

### 4.2. Overall Completeness and Applicability of Evidence

In our review, all relevant types of subjects with problems of lordosis [32,44,45] and hyperlordosis [34,35,41,42,43,46,47] were investigated. Different types of corrective programs (Lumbar stabilization exercise, Forward head posture corrective exercises, William training, Combining Core Stability with Stretching Exercises, Sling exercise, Corrective Exercises of America’s National Academy of Sports Medicine, and Pilates exercise) were recorded, but due to their number, it is not possible to do a subgroup analysis to get the effect size for each of these methods. The respondents included ranged from nine to 60 years old, which gives relevance to this review article, although for such a small number of studies involved, this may be a problem. The obtained results of our analysis give a positive message to the subjects with lordosis and hyperlordosis and related low back pain. They are proof that the problems of this type of respondent can be improved by applying for the mentioned corrective programs. Some studies have shown that the extent of lumbar lordosis is affected by age and gender, pregnancy, or obesity [20,21]. The results of our meta-analysis show that different corrective programs have a moderate effect size in older subjects (SMD = 0.520; ≥0.5 according to the Cohen guide is a moderate effect size).

### 4.3. Quality of the Evidence

All studies included together had a low risk of bias. A high risk of bias appears in the item “allocation concealment”, while the study of Cho et al. [44] was the only one to have a high risk of bias for two items. The “allocation concealment” was difficult to determine as the published reports did not provide enough details for the verdict. A pooled estimate of the magnitude of the effect was consistent, indicating the absence of heterogeneity after the exclusion of one study. Subgroup analysis shows that the magnitude of the effect of applying different corrective programs is greater in younger respondents than in older ones. The results of the subgroup analysis for the control group factor (treatment or no treatment) show uniform results. The effects of corrective programs would have been even better if the studies had used quality of life as an outcome, which assesses the subjective feelings of the respondents after the therapeutic program.

### 4.4. Potential Biases in the Review Process

We have undertaken an exhaustive search based on multiple electronic databases and supplementary sources. However, we acknowledge that other relevant studies in the gray literature or other languages may have been neglected. The decision to use one experimental group from the study of Okhli et al. [34] as a control, due to the unavailability of data from the control group, is not a bias for us because other studies report the use of different treatments in the control groups. In the study by Yakhdani et al. [41], we did not include another experimental group that used water treatment for the reason that it would be the only such group, so we hypothesized that it would lead to confusion and possibly increase heterogeneity. After performing a sensitive Leave-one-out meta-analysis, we did not exclude one of the studies, Fatemi et al. [42] or Javid et al. [43], which could be biased in the review process. The decision was made because the Leave-one-out meta-analysis showed that the results of the effect size would not change significantly (SMD = 0.550 vs. SMD = 0.535). The change would be that the number of respondents included in our analysis would be reduced from 482 participants to 442. Excluding the study, Yazici et al. [35], in our opinion, is not a bias and is a good decision, although it reduces the magnitude of the effect but solves the problem of heterogeneity. The study by Hosseinifar et al. [45] had respondents ranging from 18 to 50 years, which was a big problem in the subgroup analysis for the factor of the year, how and where to classify it, so we excluded it only for subgroup analysis, and we think that the decision was a good one. In our study, 167 male and 315 female respondents participated. Subgroup analysis by gender could not be done because some studies used mixed subjects. The use of different measuring instruments may be a bias, but this did not affect the homogeneity of the included studies. In our analysis, the nine studies included were RCT, while one study did not state whether it was randomized.

### 4.5. Agreements and Disagreements with Other Studies or Reviews

Our meta-analysis is the only one that has addressed this issue. A meta-analysis by Gálvez et al. [48] examined the effects of exercise programs on kyphosis and lordosis angle, and four studies measured LLA. The magnitude of the effect in their analysis for LLA outcome was (SMD = 0.13; according to the Cohen guide, the magnitude of the effect is negligible), with high heterogeneity (I^2^ = 80%) with the prior exclusion of one study as in our case. This difference in the effect size (SMD = 0.550 vs. SMD = 0.13), according to our assumption, lies in different degrees of freedom. We ended our analysis with 9 degrees of freedom (df = 9), and they with 3 degrees of freedom (df = 3). Therefore, there was no scope in their analysis to solve the problem of great heterogeneity. In addition to the study by Gálvez et al. [48], we did not find any more meta-analysis with which we could compare the results from our analysis. We did not comment on the values of the results from the individual studies that were included in our study because the aim of this paper was to obtain the overall magnitude of the effect size through the pooled results. 

### 4.6. Limitations and Future Studies 

This study has several limitations. First, despite a comprehensive search, our study included only those written in English. Second, despite a detailed search, the number of studies found is relatively small. We think that we should have had more coverage in the search according to the year of publication of the study. The desire was to examine studies from the last ten years. Third, the magnitude of the effect was not estimated for any outcome other than LLA.

We believe that the application of different programs in the future will lead to the fact that their magnitudes of effects can be differentiated, and better conclusions can be made about which methods would be best to use. We also recommend researchers conduct corrective programs in water, and the synthesis of their results will be useful in planning the treatment of subjects with lordosis, hyperlordosis, and related low back pain.

## 5. Conclusions

This study dealt with the effects of different corrective exercises on Lumbar lordotic angle correction in subjects with lordosis and hyperlordosis. The current meta-analysis indicates that corrective exercises have a moderately positive effect on the correction of Lumbar lordotic angles in lordotic and hyperlordotic subjects. The results realistically show the magnitude of the effect because they do not show heterogeneity. In order to arrive at such a result, however, we had to exclude the results from one study that created substantial heterogeneity. We believe that our meta-analysis can be useful to many physiotherapists and clinicians in solving problems in patients with low back pain, lordosis, and hyperlordosis, and also provide an incentive for further work and future research.

## Figures and Tables

**Table 1:** Characteristics of the included studies.

Study	N	Program Type	Outcome	Lordotic	Age	Sessions	Duration	Instrument to
				Angle		Per Week		Measure Angle
Cho 2015 [44]	30	Lumbar stabilization exercise	LLA	32.1° ± 3.3°	48 ± 6.9	(3× week)	6 weeks	Plain
		Conservative treatment		32.3° ± 3.5°	44 ± 6.7			radiography
Fatemi 2015 [42]	40	William training	LLA	55.22° ± 6.00°	15–18	(3× week)	8 weeks	Flexible ruler
		Control group (no treatment)		54.48° ± 6.54°				
Hosseinifar 2018 [45]	32	Stabilization exercise	LLA	27.63° ± 3.3°	18–50	(6× week)	2 weeks	Flexible ruler
		Control group (physiotherapy protocol)		27.63° ± 4.7°				
Javadipour 2020 [46]	30	Stretching-core stability exercises	LLA	62.25° ± 3.88°	13–15	(3× week)	8 weeks	Flexible ruler
		Control group (no treatment)		62.25° ± 3.88°				
Javid 2014 [43]	40	William training	LLA	55.22° ± 6.00°	15–18	(3× week)	8 weeks	Flexible ruler
		Control group (no treatment)		54.48° ± 6.54°				
Ko 2018 [32]	29	Lumbar stabilization exercise	LLA	40.0° ± 1.5°	43.1 ± 3.7	(3× week)	12 weeks	ViewRex
		Sling exercise		38.9° ± 2.1°	43.6 ± 4.5			system
		Control group (no treatment)		40.0° ± 2.4°	41.3 ± 3.8			
Moustafa 2015 [47]	154	Corrective exercises	LLA	49.5° ± 3.4°	49.1 ± 4.9	(2× week) first 4 weeks	10 weeks	Formetric
		Control group (standard care)		49.3° ± 3.2°	50.5 ± 4.8	(3× week) second 6 weeks		system
Okhli 2019 [34]	45	NASM	LLA	53.7° ± 1.83°	15.26 ± 1.03	(3× week)	8 weeks	Flexible ruler
		Pilates exercises		53.13° ± 1.73°	15.02 ± 1.28			
		Control group (no treatment)		N/A	14.76 ± 1.13			
Yakhdani 2017 [41]	42	Water exercises	LLA	68.43° ± 11.75°	9–12	N/A	8 weeks	Flexible ruler
		Land exercises		63.18° ± 11.5°				
		Control group (no treatment)		61.32° ± 6.43°				
Yazici 2017 [35]	40	Corrective exercise	LLA	48.23° ± 1.74°	16.84 ± 1.84	(3× week)	8 weeks	Spinal Mouse
		Control group (no treatment)		48.51° ± 2.18°				device

LLA—Lumbar lordotic angle; N/A—no answer; NASM—National Academy of Sports.

## Data Availability

Not applicable.
